# No difference in overall survival for primary cutaneous Merkel cell carcinoma after Mohs micrographic surgery and wide local excision: A multicenter cohort analysis

**DOI:** 10.1016/j.jdin.2024.03.001

**Published:** 2024-04-04

**Authors:** Ajay N. Sharma, Addison M. Demer

**Affiliations:** a Department of Dermatology, University of California Irvine, Irvine, California; b Department of Dermatology, Mayo Clinic, Rochester, Minnesota

**Keywords:** bleeding, complications, disparities, hematoma, infection, Kaplan-Meier, Merkel cell carcinoma, Mohs, Mohs surgery, outcomes, sentinel lymph node, survival, wide local excision

*To the Editor:* Merkel cell carcinoma (MCC) remains among the most dangerous cutaneous cancers, with a propensity for distant metastases and a strikingly high associated mortality.^1^ Treatment has historically relied on wide local excision (WLE), but Mohs micrographic surgery (MMS) remains an alternative surgical technique that allows for flexible surgical margins by utilizing complete intraoperative margin assessment.^2^ To compare both techniques, this study analyzes clinical outcomes of patients with primary cutaneous MCC through a national, deidentified health care database.

A global health research network (TriNetX) consisting of medical records from 100 health care organizations was used to identify cohorts of patients with primary cutaneous MCC treated with either WLE or MMS. Prior to assessing outcomes, both cohorts were propensity score–matched for age, sex, race, American Joint Committee on Cancer staging, and adjuvant treatment (chemoimmunotherapy and/or radiation). International Classification of Diseases 10th Revision codes were then used to identify overall survival and treatment complications, including infection, bleeding, and hematoma rate.

A total of 291 patients were treated with MMS, whereas a total of 1368 patients were treated with WLE (Table I). Patient age averaged approximately 75 years, with 71% being men and 93% being White. A similar proportion of the WLE group underwent sentinel lymph node biopsy compared with the MMS group (29% vs 26%; *P* = .40). After matching for age, sex, race, American Joint Committee on Cancer tumor stage, and adjuvant treatment, outcome analysis demonstrated that patients who underwent MMS had no statistically significant difference in overall survival at 1, 3, 5, or 10 years compared with those treated with WLE (Fig 1). Minimal differences were seen with surgical complications in the perioperative period. Analysis revealed no difference in 30-day surgical site infection rate (3.5% vs 5.6%; *P* = .22) and postoperative bleeding rate (3.5% vs 3.5%; *P* = 1) between both cohorts. There was a small disparity in postoperative hematoma complication rate (3.5% vs 0%; *P* < .01) in the MMS group compared with the WLE group.

**Table I:** Comparison of Mohs micrographic surgery and wide local excision cohorts and outcomes, before and after propensity score matching, for the treatment of Merkel cell carcinoma

	Before matching			After matching		
	MMS	WLE	*P* value	MMS	WLE	*P* value
No. of patients	291	1368		286	286	
Age (y), mean ± SD	75.4 ± 9.41	74.5 ± 10.2	.15	75.5 ± 9.4	76.0 ± 10.1	.54
Sex, *n* (%)						
Male	208 (71%)	839 (72%)	**< .05**	203 (71%)	199 (71%)	.71
Female	83 (29%)	489 (28%)	**< .05**	83 (29%)	87 (29%)	.71
Race, *n* (%)						
White	270 (93%)	1232 (90%)	.16	266 (93%)	265 (93%)	.87
Unknown	10 (3%)	13 (1%)	**< .01**	10 (3%)	10 (3%)	1
American Indian	10 (3%)	10 (1%)	1	10 (3%)	10 (3%)	1
Asian	10 (3%)	17 (2%)	**< .01**	10 (3%)	10 (3%)	1
Black	10 (3%)	10 (1%)	1	10 (3%)	10 (3%)	1
Stage, *n* (%)						
AJCC 0	10 (3%)	10 (1%)	**< .01**	10 (3%)	10 (3%)	1
AJCC 1	20 (7%)	76 (6%)	**< .01**	19 (7%)	12 (4%)	.19
AJCC 2	10 (3%)	55 (4%)	**< .01**	10 (3%)	14 (5%)	.40
AJCC 3	10 (3%)	72 (6%)	**< .01**	10 (3%)	14 (5%)	.40
AJCC 4	10 (3%)	10 (1%)	**< .01**	10 (3%)	10 (3%)	1
Complications, *n* (%)						
Infection (30-d)	10 (3.4%)	46 (3.4%)	.95	10 (3.5%)	16 (5.6%)	.22
Bleeding (30-d)	10 (3.4%)	10 (0.7%)	**< .05**	10 (3.5%)	0 (0%)	**< .01**
Hematoma (30-d)	10 (3.4%)	16 (1.1%)	**< .05**	10 (3.5%)	10 (3.5%)	1
Overall survival, *n* (%)						
Mortality (1-y)	28 (9.6%)	148 (10.8%)	.54	27 (9.4%)	32 (11.1%)	.49
Mortality (3-y)	69 (23.7%)	287 (21.0%)	.30	68 (23.8%)	75 (26.2%)	.83
Mortality (5-y)	87 (29.9%)	353 (25.8%)	.15	85 (29.7%)	90 (31.5%)	.65
Mortality (10-y)	103 (35.4%)	392 (28.7%)	**< .05**	101 (35.3%)	100 (35.0%)	.93

Bolded *P*-values: < .05 or < .01.

*AJCC*, American Joint Committee on Cancer; *MMS*, Mohs micrographic surgery; *WLE*, wide local excision.

**Fig 1:**
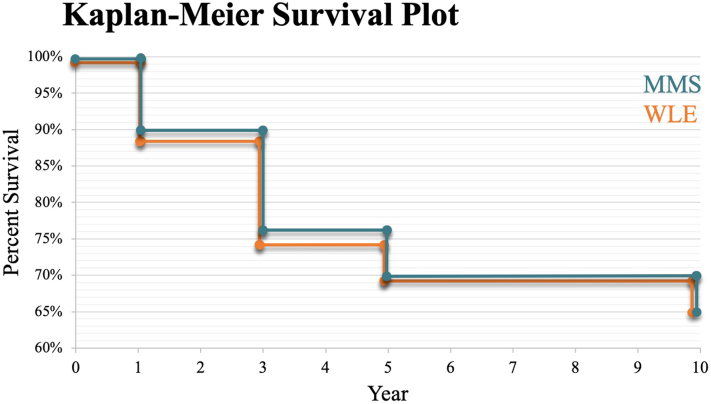
Comparing overall survival from 0 to 10 years after Mohs micrographic surgery (MMS; green) and wide local excision (WLE; orange) for Merkel cell carcinoma after propensity score matching.

These data support MMS as an effective surgical treatment option for MCC, perhaps highlighting the benefit of complete histologic clearance over predetermined surgical margins. Patients who underwent MMS had no difference in outcomes compared with those who underwent WLE in overall survival for all stages for up to 10 years, concordant with recent literature.^3^^,^^4^ Similarly, these results suggest relatively equal morbidity with MMS, with the same infection and bleeding rates, as well as a minimal difference in hematoma rate. Given that the treatment of MCC frequently requires sentinel lymph node biopsy, adjuvant radiation, and/or adjuvant chemoimmunotherapy, equivalent outcomes with MMS, which also possesses advantages in tissue conversation, may warrant further consideration of this surgical technique for select tumors.^5^

TriNetX is a hospital-based registry that does not report certain clinicopathologic variables (eg, overall survival stratified by stage) or may underreport others (eg, sentinel lymph node biopsy), leading to limitations. Although multivariable analysis controlled for various factors associated with overall survival, disease-specific survival and local recurrence rate are arguably more relevant for the evaluation of MCC. With these patients most often of elderly age, the cause of death may be due to reasons unrelated to MCC entirely.

## Conflicts of interest

None disclosed.
